# Studying Huntington’s Disease in Yeast: From Mechanisms to Pharmacological Approaches

**DOI:** 10.3389/fnmol.2018.00318

**Published:** 2018-09-04

**Authors:** Sebastian Hofer, Katharina Kainz, Andreas Zimmermann, Maria A. Bauer, Tobias Pendl, Michael Poglitsch, Frank Madeo, Didac Carmona-Gutierrez

**Affiliations:** ^1^Institute of Molecular Biosciences, University of Graz, Graz, Austria; ^2^Department of Internal Medicine, Medical University of Graz, Graz, Austria; ^3^BioTechMed-Graz, Graz, Austria

**Keywords:** Chorea Huntington, huntingtin, drug discovery, disease model, neurodegeneration, apoptosis, aging

## Abstract

Huntington’s disease (HD) is a neurodegenerative disorder that leads to progressive neuronal loss, provoking impaired motor control, cognitive decline, and dementia. So far, HD remains incurable, and available drugs are effective only for symptomatic management. HD is caused by a mutant form of the huntingtin protein, which harbors an elongated polyglutamine domain and is highly prone to aggregation. However, many aspects underlying the cytotoxicity of mutant huntingtin (mHTT) remain elusive, hindering the efficient development of applicable interventions to counteract HD. An important strategy to obtain molecular insights into human disorders in general is the use of eukaryotic model organisms, which are easy to genetically manipulate and display a high degree of conservation regarding disease-relevant cellular processes. The budding yeast *Saccharomyces cerevisiae* has a long-standing and successful history in modeling a plethora of human maladies and has recently emerged as an effective tool to study neurodegenerative disorders, including HD. Here, we summarize some of the most important contributions of yeast to HD research, specifically concerning the elucidation of mechanistic features of mHTT cytotoxicity and the potential of yeast as a platform to screen for pharmacological agents against HD.

## Introduction

Since the first detailed and widely recognized description of Huntington’s disease (HD) by George Huntington in 1872 (Hayden, 1981, 1983), there have been important advances in understanding this malady. However, to date, the underlying molecular mechanisms as well as the cellular and organismal perturbations that occur in HD patients are not fully understood (Reiner et al., 2011; Kumar et al., 2015). In fact, there is still no cure for the disease, and therapeutic approaches are only limited to symptomatic treatments. It is therefore a pending issue to elucidate the causal network behind this clinically well characterized disease (Roos, 2010) to eventually develop therapies that can revert and/or prevent HD. The application of adequate model systems allows for a useful and rapid possibility to tackle both basic mechanistic research and the explorative quest for new therapeutics. In this review, we will summarize the use and potential of the budding yeast *Saccharomyces cerevisiae* as a model organism in HD research. Since manifold reviews have already been published on the topic (Bates and Hockly, 2003; Coughlan and Brodsky, 2005; Outeiro and Giorgini, 2006; Winderickx et al., 2008; Giorgini and Muchowski, 2009; Duennwald, 2011; Mason and Giorgini, 2011; Pereira et al., 2012; Tenreiro et al., 2013; Peffer et al., 2015; Shrestha and Megeney, 2015; Fruhmann et al., 2017), in this work, we will specifically address critical limitations and delineate the translational power of this tool for both mechanistic insights and drug discovery. In particular, we will focus on discussing studies that highlight the translational and human-relevant potential harbored by HD research conducted in *S. cerevisiae*.

### Huntington’s Disease

Huntington’s disease is one of nine CAG trinucleotide repeat disorders, a group of inherited neurodegenerative diseases (Zoghbi and Orr, 2000; Nakamura et al., 2001) and is caused by a CAG repeat (codes for glutamine, Q) mutation in exon-1 of the huntingtin (HTT) gene, which results in an elongated polyglutamine [or poly(Q)] stretch of the protein – giving rise to the term mutant HTT (mHTT) (McColgan and Tabrizi, 2017). HD is most prevalent in European societies and can nowadays be diagnosed with reliable genetic tests. In European populations, the HD mutation carrier rate is roughly 10.6 - 13.7/100,000 (Walker, 2007; Bates et al., 2015). The disease is inherited in an autosomal dominant manner and its onset occurs at an average age of 45 years with an asymptomatic, subtle, pre-clinical phase preceding the actual clinical stage by up to 15 years (Ross et al., 2014). Cases of hereditary HD can be traced back for several generations, while in rare cases spontaneous occurrence of non-inherited HD can appear due to genetic instability in parents with CAG repeats, whose number is at the high-end of the normal range (up to 35 repeats, see below) (Shaw and Caro, 1982). In fact, it is now believed that up to 10–15% of HD cases are of spontaneous nature (Reiner et al., 2011). Clinical symptoms include cognitive impairments, motor dysfunction, emotional disturbance, neuropsychiatric alterations, and striatal atrophy, ultimately leading to premature death (Walker, 2007). Many of these symptoms were already described by George Huntington and others in the 19th century (Hayden, 1981, 1983). However, despite huge progress in understanding the disease, there exist no causal treatments yet. Most experimental therapeutic approaches aim at reducing the load of the mutant protein, with the so-called anti-sense oligonucleotide therapy being among the most promising against HD. This multifaceted technique employs allele-specific or -unspecific oligonucleotides targeting HTT DNA or mRNA (Zaghloul et al., 2017). This appealing approach has already been successfully employed in the well-established YAC128 mouse model of HD (Stanek et al., 2013). Of note, recent findings have revealed significant neurotoxic potential of mutant HTT (mHTT)-derived small and elongated RNA species, which may be trapped in the nucleus and compromise cellular viability (de Mezer et al., 2011; Bañez-Coronel et al., 2012).

Healthy individuals possess a CAG count below 35 repeats, whereas HD patients harbor an HTT gene with longer poly(Q) tracts: full penetrance of the disease is believed to occur at 40 or more repeats, whereas poly(Q) regions of 36–39 repeats lead to reduced penetrance, with a chance of no, or very late onset of the disease. Thus, in humans, as well as in model organisms, disease onset (and severity to some extent) seems to correlate with the length of the poly(Q) tract (Snell et al., 1993; Penney et al., 1997; Adegbuyiro et al., 2017). Other (genetic) factors, however, modulate HD as well, but cannot compensate for the causal role of elongated poly(Q) regions within the HTT protein (MacDonald et al., 1993; Finkbeiner, 2011; Reiner et al., 2011). These elongations result in structural alterations, which subsequently make the protein prone for misfolding, aggregation, and fragmentation, leading to intranuclear and cytosolic multi-protein inclusion bodies in brain cells (Davies et al., 1997; Bates et al., 2015), which disturb cellular homeostasis. It is a matter of intense debate whether (large) HTT protein aggregates are the toxic elements or represent a detoxified (or even protective) form. This discrepancy and the importance of different intermediate oligomers would go beyond the scope of this review and will not be discussed here in detail, but definitely asks for clarification in the future (Arrasate and Finkbeiner, 2012). Adding to the disease’s complexity, HTT is ubiquitously expressed and, contrary to earlier paradigms, more recent advances highlight the fact that HD affects the human body not only at the neuronal, but also at a systemic level (van der Burg et al., 2009).

The cloning of the HTT gene in 1993 led to a boost in HD research. IT-15 (interesting transcript 15) on chromosome four was identified by “The Huntington’s Disease Collaborative Research Group” and shown to contain an unstable (CAG) region that is essential for the toxic role of the protein, followed by an adjacent proline-rich region (PRD) (MacDonald et al., 1993). The discovery of a distinct protein (HTT) at the heart of this neurodegenerative disease also sparked the development of genetic models in various vertebrate (rhesus monkey, sheep, Tibetan miniature pig, rodents, zebrafish), invertebrate (*Drosophila melanogaster*, *Caenorhabditis elegans*), and unicellular (*S. cerevisiae*) organisms (Mason and Giorgini, 2011; Xi et al., 2011; Morton and Howland, 2013; Pouladi et al., 2013). In addition to animal- and unicellular-based HD models, genetically tractable *in vitro* cell culture models have been helpful in deducing the molecular basis of neurodegenerative diseases. Especially in recent years, advances in a technique that employs “induced pluripotent stem cells” (iPSCs) are growingly becoming useful and iPSCs have been applied to HD research already since 2008 [Park et al., 2008; reviewed in Tousley and Kegel-Gleason (2016)]. Nowadays, a wide range of different iPSC lines are available, recapitulating most of the pathological features on a cellular scale as seen in human patients and other models (Tousley and Kegel-Gleason, 2016). These iPSC human cell lines will be of great importance, as they allow time- and cost-efficient screens for drug discovery (Tousley and Kegel-Gleason, 2016). Still, tackling the wide range of significant questions related to HD in the most rapid manner will require a combinatory approach that uses all relevant information using different models at hand. For that, in the scope of this review, we will only discuss the usability and limitations of unicellular yeasts as HD models.

The 67 exons of the HTT gene encode a ∼350-kDa protein, including the polymorphic trinucleotide (CAG) track within exon-1 (MacDonald et al., 1993). With the presence of homologs in many species (but not yeast), HTT is believed to have pleiotropic, potentially conserved molecular and physiological functions (Reiner et al., 2011). Interactors of the ubiquitously expressed HTT have been described in the range of 40 to several hundred proteins, and the list of suggested native functions is long, including its involvement in autophagy, transcriptional regulation, endocytosis, vesicle function, endosomal trafficking, cell division, ciliogenesis, and antiapoptotic processes (Schulte and Littleton, 2011; Saudou and Humbert, 2016). In fact, while the non-physiological gain-of-function mutation phenotype of HTT is the basis for most HD research in model organisms (Bates and Hockly, 2003; Bates et al., 2015), it does not fully account for the molecular pathologic events seen in human HD cells. A loss-of-function toxicity of *physiological* HTT-tasks likely contributes to the observed detrimental phenotypes (Zuccato and Cattaneo, 2014; Bates et al., 2015).

Among the molecular pathologic features of HD are excessive misfolding, aggregation, transcriptional dysregulation, mitochondrial dysfunction, and altered energy metabolism, impairments of the proteasomal and autophagic system, and many more (Roos, 2010). Intriguingly, using a fruitfly model of HD, Ochaba et al. (2014) have recently discovered a housekeeping function of HTT as a scaffold protein in selective autophagy. Selective autophagy is a catabolic process important for the clearance of intracellular content and is heavily implicated in neurodegenerative diseases (Wong and Cuervo, 2010; Johansen and Lamark, 2011). This discovery also holds evidence for a possible two-faceted pathological role of HTT in HD: the mutated protein is likely to be toxic *per se*, but also titrates functional HTT protein, deriving in impaired autophagic competence and a cascade of cellular perturbations (Ochaba et al., 2014).

Adding to the complex molecular features of HD, more recent reports have shed light on different RNA-based toxicity mechanisms. Seemingly, mHTT transcription can lead to short repeated CAG RNA species and nuclear RNA inclusions. Nucleolar stress, alterations in alternative splicing processes, disruption of nuclear functions by binding of diverse factors to elongated repeats, and cellular dysfunction may be the consequences thereof (Martí, 2016). Moreover, so-called RAN (repeat-associated non-ATG) peptides seem to contribute to HD pathology and might comprise another molecular source of mHTT toxicity (Bañez-Coronel et al., 2015).

This combination of increased lethal and decreased vital effects of HTT increases the difficulty of drug development, as both physiological and pathological functions of HTT have to be taken into consideration. Importantly, as opposed to other HD models, the yeast *S. cerevisiae* represents a “clean” cellular room (since it has no HTT homolog) that, however, possesses a conserved regulated cell death (RCD) machinery. This makes yeast a valuable addition to available higher eukaryotic HD models.

### Yeast as a Model Organism for Neurodegenerative Diseases

The budding yeast *S. cerevisiae* is a unicellular eukaryote that has accompanied human societies and culture since the distant past. In more recent times, yeast has additionally become an indispensable and strikingly well-characterized tool in (food) industry and research. In fact, it is considered the best studied eukaryotic cell system with over 6,000 known protein-coding genes, which have largely been annotated. A major advantage for early yeast researchers was the fully sequenced genome, published in 1996, and thereafter, the large number of available systematic studies at the genome and proteome levels (Goffeau et al., 1996; Botstein and Fink, 2011; Engel et al., 2013). Only recently, the Nobel Prize in Physiology or Medicine 2016 was awarded to Yoshinori Ohsumi for his work on autophagy, mainly performed in yeast. Of note, this has been the fifth Nobel prize awarded to work performed with yeast in the 21st century (Zimmermann et al., 2016).

Yeast is widely used as a versatile model organism (Botstein and Fink, 2011), including but not limited to the research fields of RCD (Madeo et al., 1999, 2002; Büttner et al., 2011; Munoz et al., 2012; Côrte-Real and Madeo, 2013; Fahrenkrog, 2015; Carmona-Gutierrez et al., 2018), systems biology (Mustacchi et al., 2006), aging (Gershon and Gershon, 2000; Fröhlich and Madeo, 2001; Herker et al., 2004; Kaeberlein et al., 2007; Janssens and Veenhoff, 2016), prion biology (Speldewinde and Grant, 2015, 2017), virus research (Lista et al., 2017; Zhao, 2017), genetics (Forsburg, 2001; Hagihara et al., 2017), autophagy (Reggiori and Klionsky, 2013; Ohsumi, 2014; Yin et al., 2016) as well as of neurodegeneration (Khurana and Lindquist, 2010; Tenreiro and Outeiro, 2010; Ocampo and Barrientos, 2011; Pereira et al., 2012; Shrestha and Megeney, 2015; Stekovic et al., 2017), drug discovery, and drug development (Ma, 2001; Hughes, 2002; Outeiro and Giorgini, 2006; Eisenberg et al., 2009, 2016; Amen and Kaganovich, 2016; Madeo et al., 2018; Zimmermann et al., 2018). Yeast experiments are usually low cost and can be performed in a high-throughput manner. This makes it suitable for large-scale genetic and pharmacological screens. Yeast researchers can draw from rich sources of freely available databases (e.g., SGD, CYGD, yGMV), extensive knock-out, overexpression, and fusion protein strain collections (e.g., EUROSCARF), handy genetic tools and a large research community (Khurana and Lindquist, 2010; Tenreiro and Outeiro, 2010).

Overall, *S. cerevisiae* provides all the features and techniques needed for a valid model organism. To date, no other yeast species has reached a similar degree of research usability. However, in the future, other yeast-based models may become increasingly important for HD research, especially *Schizosaccharomyces pombe* and *Pichia pastoris*. *S. cerevisiae* has traditionally emerged as the most wide-spread yeast model of choice, since it is highly flexible as an organism and has proven to be a valid tool in other neurodegenerative diseases. It is one of the most robust *in vivo* tools for high-throughput drug- and mechanism-screenings. Moreover, *S. cerevisiae* is at the cutting edge of systems biology, with techniques and tools to generate and analyze “big data” ready to be employed. The combination of genetic manipulations with flexible experimental designs and short experimental durations allows researchers to rapidly approach scientific questions and manage research schedules that are not feasible in other model organisms. This tremendous wealth of resources has led to a vast scientific output from studies conducted in this simple eukaryote.

Besides other disorders, tractable *S. cerevisiae* models have been developed and intensively studied for neurodegenerative diseases such as HD (see following paragraphs), Alzheimer’s disease (Heinisch and Brandt, 2016; Verduyckt et al., 2016), Parkinson’s disease (Menezes et al., 2015; Tenreiro et al., 2017), Friedreich’s Ataxia (Babcock et al., 1997), Batten’s disease (Pearce and Sherman, 1998; Rajakumar et al., 2017), amyotrophic lateral sclerosis (Johnson et al., 2008; Kryndushkin and Shewmaker, 2011; Leibiger et al., 2018), Niemann-Pick disease (Berger et al., 2005; Rajakumar et al., 2017), and others (Tenreiro and Outeiro, 2010; Pereira et al., 2012; Ring et al., 2017). Importantly, a variety of genes (>1,000) related to human diseases have orthologs in the yeast genome, many of which can be genetically and functionally replaced by their human counterparts (Menezes et al., 2015). The development of so-called “humanized” yeast strains by modifying yeast genes or replacing whole pathways to resemble human cell biology (Kachroo et al., 2015; Laurent et al., 2016; Truong and Boeke, 2017) has added to the importance of this model organism and redefined its role for human-relevant research (Kachroo et al., 2015; Laurent et al., 2016). Although yeast is regarded as a unicellular organism, yeast cells harbor many of the cellular pathways needed for metabolic homeostasis and protein quality control that have been implicated in neurodegeneration (Barrientos, 2003; Ocampo and Barrientos, 2008), including RCD subroutines (Carmona-Gutierrez and Büttner, 2014; Carmona-Gutierrez et al., 2018) and autophagic processes (Zimmermann et al., 2016; Kainz et al., 2017). Furthermore, yeast cells have been shown to interact with each other in complex manners under specific physiological conditions. This has prompted to overcome the classical dogma of yeast populations being a mere accumulation of single independent elements and to reinterpret them as multicellular entities that undergo intercellular communication, ensuring the survival of the clone (Honigberg, 2011; Carmona-Gutierrez and Büttner, 2014). This may further explain the amount of scientific questions with relevance to humans that can be addressed in yeast.

In summary, *S. cerevisiae* represents the yeast species of choice for a manifold of scientific questions. To date, no other yeast has reached a similarly multilayered level of usability. Nonetheless, it can be expected that the future will bring a wider range of HD models in various yeasts, which will then add to the richness of scientific and technical possibilities.

## Modeling HD in Yeast

Despite the high degree of conservation of human disease-associated genes in yeast, S. cerevisiae – as mentioned above – lacks HTT homologs. Therefore, yeast HD models are based on the heterologous expression of human HTT in various versions (**Table 1**). Noteworthy, N-terminal HTT fragments are an intrinsic feature of human HD pathology (Davies et al., 1997) and many yeast HTT-poly(Q) constructs are modeled accordingly, mostly using HTT exon 1. Different models of mHTT will be discussed in the following chapters, using the term “mHTT” as an identifier for variants with elongated poly(Q) tracts. While the pathological threshold for the poly(Q) length has been well studied in humans, as far as we know, it is not known in yeast. Thus “mHTT” mostly refers to constructs with poly(Q) lengths well above the threshold in humans.

**Table 1:** Overview of important yeast HD models and their basic characteristics.

Original and significant publications	Organism	Promotor (Inducer)	Poly(Q) Lengths	Integrated (i)/Plasmid (p)	N-terminal HTT aminoacids	Cytotoxicity	Aggregation
Krobitsch and Lindquist, 2000	*S. cerevisiae*	*GAL1* (galactose)	25, 42, 72, 103	p	68	No	Yes
Muchowski et al., 2000	*S. cerevisiae*	*CUP1* (copper)	20, 39, 53	p	? (exon-1)	No	Yes
Meriin et al., 2002, 2003	*S. cerevisiae*	*GAL1* (galactose)	25, 103	p	17	Yes	Yes
Solans et al., 2006	*S. cerevisiae*	*GAL1* (galactose)	25, 103	i and p	17	Yes	Yes
Ocampo and Barrientos, 2008; Ruetenik et al., 2016	*S. cerevisiae*	*TEF1-7* (β-estradiol)	25, 46, 72, 103	p	17	Yes	Yes
Kaiser et al., 2013; Papsdorf et al., 2015	*S. cerevisiae*	*GPD* (constitutive)	0, 30, 56	p	0	Yes	Yes
Zurawel et al., 2016	*S. pombe*	*P3nmt1* (thiamine repressible)	25, 46, 72, 103	i	? (exon-1)	No	Yes


### Non-toxic Models

Krobitsch and Lindquist (2000) established a poly(Q)-length-dependent model of HTT aggregation in 2000 by fusing the first 68 N-terminal amino acids of wild-type HTT exon-1 containing poly(Q) tracts of varying length (25, 42, 72, 103 glutamines) to a C-terminal GFP (green fluorescent protein)-tag. For stability reasons, they modified the codons within the poly(Q) tract to a mixture of CAG and CAA. They could either rule out or confirm the involvement of various specific chaperones in the aggregation process. Notably, observed effects were consistent for different low/high-copy plasmids with constitutive or inducible (galactose-driven) expression. Remarkably, deletion of HSP104 completely abolished aggregation of Htt103Q. However, this early model did not show cytotoxicity. Later studies revealed a strong dependency of mHTT toxicity on the flanking sequences (Dehay and Bertolotti, 2006; Duennwald et al., 2006a; Jiang et al., 2016), which might explain the lack of cytotoxicity in the model used by Krobitsch and Lindquist (2000). Interestingly, shortly after this study, it was shown that in a similar model redirecting fragmented exon-1-poly(Q)-fusion proteins to the nucleus, nuclear aggregates are formed, which are unaffected by HSP104 deletion (Cao et al., 2001). From this observation, it can be hypothesized that aggregation of these HTT fragments in yeast is indeed a controllable process and that different mechanisms exist between the nucleus and the cytoplasm. These early reports, indicating crucial roles of the chaperone system in yeast HD cytotoxicity, were then further validated and confirmed in numerous other studies (see below).

### Classical Models of HD in *S. cerevisiae* and the Implication of Prions

Also, Muchowski et al. (2000) cloned human HTT exon-1 into the high copy Yep105 vector (*CUP1* promoter, copper-inducible) containing 20, 39, or 53 glutamines, respectively. Coexpression of the chaperones Hsp40 (yeast Ydj1) and Hsp70 (yeast Ssa1 but, conversely, not Ssb1) prevented the formation of large Htt53Q aggregates in favor of smaller, SDS-soluble aggregate forms (Muchowski et al., 2000). The lack of an effect (or at least a greatly diminished effect) seen for Ssb1p coexpression could be explained by the different functions of the two members of the Hsp70 family: Ssa1p acts posttranslationally (Bush and Meyer, 1996), whereas Ssb1p associates to newly formed proteins at the ribosomes (Nelson et al., 1992; Pfund et al., 1998). In a follow-up study, the same model was employed in a genome-wide synthetic-lethal screen, elucidating important roles of protein folding and stress response processes in HD. Consequently, they found that the Hsp40 chaperone genes *APJ1* and *HLJ1* seem to be necessary to suppress mHTT toxicity. Interestingly, the authors could not find correlations between toxicity and aggregation levels (Willingham et al., 2003).

One of the nowadays most extensively used models was developed by Meriin et al. (2002). It was constructed by cloning the first 17 amino acids of HTT exon-1 followed by 25 or 103 glutamines into a pYES2-vector (*GAL1* promoter, galactose-inducible). The sequences were N- and C-terminally flanked by a FLAG- and GFP-tag, respectively. In their original publication, they could show that Htt103Q accumulates in and is toxic to yeast cells. Furthermore, they observed that molecular chaperones were involved in the aggregation process and that toxicity and aggregation were linked in a correlative manner. Moreover, deletion of the prion gene *RNQ1* abolished poly(Q) toxicity. The authors further concluded that *HSP104*, which is also linked to prion formation, was crucially involved in the nucleation of newly formed aggregates, but not in their growth (Meriin et al., 2002). Of note, in Δ*hsp104* strains spreading of prions is diminished, but its overexpression can conversely also modulate prion formation and/or propagation (Jones and Tuite, 2005). Using the same model, Meriin et al. (2003, 2007) went on to study the fate of endocytosis in mHTT-expressing yeast cells and shed light on the recruitment of proteins into the malignant intracellular aggregates. To circumvent expression problems during long experiments (e.g., chronological aging) when using galactose-inducible systems, possibly arising due to plasmid loss, Solans et al. (2006) used the Meriin model and cloned both Htt25Q- and Htt103Q-fusion proteins into the episomal Yep351 and integrative Yip351 plasmids, again controlled by the *GAL1* promoter. The authors described the detrimental impact of Htt103Q on mitochondria and the possibilities to counteract that (Ocampo et al., 2009).

### Ends Matter – The Flanking Regions of the Poly(Q) Tract Determine Aggregation and Toxicity

The flanking regions of mHTT exon-1 seem to have a determinant impact on its toxicity. At least in yeast, poly(Q) toxicity of a human exon-1 fusion construct, as used in most models, is largely dependent on the flanking amino acid sequences. Changes in these flanking regions consequently affect both aggregation and cytotoxicity. The native HTT protein harbors a poly-proline region (poly(P)) at the C-terminus of the poly(Q) tract. It has been suggested that this poly(P) region might have evolved as a protective feature that increases the threshold of Q-repeats for aggregation (Darnell et al., 2007). In line, Htt103Q lacking the neighboring poly(P) region led to multiple small intracellular aggregates, aggravated toxicity (Dehay and Bertolotti, 2006; Duennwald et al., 2006a) and increased the growth defect of Htt103Q-expressing cells (Walter et al., 2014). Furthermore, the poly(P) region interacts with type-1 myosins and, intriguingly, in endocytosis-deficient (e.g., Δ*myo5*) cells, the aggregate-prone form Htt103Q is not toxic, while the non-toxic poly(Q) lengths can display significant toxicity. This might represent an explanation for the positive selection of elongated poly(Q)-tracts (Berglund et al., 2017).

Likewise, in yeast, a FLAG-tag – independent of its position at either the N- or C-terminus – is required for Htt103Q toxicity (at least in the W303 strain) (Duennwald et al., 2006a). Of note, replacement of the FLAG-tag with a similar-sized HA-tag did not produce the same growth defects caused by Htt103Q (Duennwald et al., 2006a). The authors of that study speculated that the negatively charged FLAG-tag mimics sumoylation, a feature of human HD and a modulator of toxicity in some models (Steffan et al., 2004).

Very similar results were obtained *in vitro* and in mammalian neuronal cell culture, showing that the importance of flanking regions for mHTT toxicity is not a yeast-specific artifact. Apparently, both N- and C-terminal regions play an important role in mediating the aggregation dynamics, with the N-terminal amino acids favoring amyloid structures and the C-terminal poly(P) sequence slowing the process (Bhattacharyya et al., 2006; Crick et al., 2013). In fact, these *in vitro* observations were confirmed in mammalian neuronal cells, revealing that the poly(P) region ameliorated aggregation and amyloid fibrillation, whereas the 17 N-terminally flanking amino acids promoted it (Shen et al., 2016). Interference with either region can, thus, drastically influence aggregation behavior, phenotypes, and toxicity.

In addition, both the FLAG-tag and the poly(P) region can mediate their effects *in trans*, and other glutamine-rich proteins may affect poly(Q) toxicity (Duennwald et al., 2006b). This strongly argues for protein interaction and recruitment to poly(Q) proteins or aggregates as an important step toward cytotoxicity. Of note, introducing poly(Q) sequences into unrelated genes can render proteins prone for aggregation (Ordway et al., 1997), suggesting that poly(Q) expansions alone hold important neurodegenerative features.

Importantly, yeast-optimized C-terminal fluorescent proteins (FPs) also affect phenotypes caused by exon1-Htt103Q expression (Jiang et al., 2016). The presence of an FP was essential for elongated poly(Q) phenotypes *per se*, and both aggregation patterns and toxicity were altered by different FPs. From the tested FPs, the blue FP yomTagBFP2 prevented Htt72Q aggregation and toxicity, probably via its strong tendency to self-assemble into oligomers, which could prevent mHTT-specific inclusion body formation (Jiang et al., 2016). Altogether, flanking regions can profoundly modulate cytotoxicity in yeast and other systems (Lakhani et al., 2010). Therefore, their crucial impact on toxic mechanisms must be considered upon working with yeast models of HD.

### Recent Advantages in Modeling HD in Yeast

Ocampo and Barrientos (2008) introduced an alternative system, conveniently eliminating the need for the metabolically active galactose as an acute inductor and allowing to control expression both in time and strength by making use of the β-estradiol-inducible *TEF1-7* promoter. This flexible system has a very tight expression control at various growth stages without the need to change the carbon source. Using this system with fragmented exon-1 containing 25, 46, 72, or 103 glutamines, the group studied the mitochondrial role in mHTT toxicity and could translate basic concepts of their findings into a Drosophila model of HD (Ruetenik et al., 2016).

Yet another model was recently developed to study ploidy control and mitochondrial dysfunction (Kaiser et al., 2013; Papsdorf et al., 2015). The authors used a length-dependent model of mHTT toxicity by cloning poly(Q)-tracts of 0, 30, and 56 glutamines into a p425GPD vector [which harbors the strong constitutive *GPD* (glyceraldehyde-3-phosphat dehydrogenase) promoter], preserving the plasmid’s C-terminal polylinker region and the eYFP gene (Kaiser et al., 2013).

### Models of HD in Non-*Saccharomyces* Yeasts

Being the best and longest studied yeast model organism, *S. cerevisiae* has gained the most attention from researchers studying HD and other neurodegenerative diseases. To date, we are not aware of any other significant yeasts being extensively employed as HD models. However, Zurawel et al. (2016) developed an integrated, thiamine-repressible (P3nmt1 promoter) system, tagged with FLAG (N-terminal) and GFP (C-terminal) in *Schizosaccharomyces pombe*. They observed mainly cytoplasmic aggregation with only a small percentage of nuclear aggregates in Htt103Q-expressing cells. This was accompanied by mild growth defects for Htt72Q and Htt103Q. Notably, while in *S. pombe*, the poly(Q) stretch seemed to be genetically stable, the authors also showed that in *S. cerevisiae*, the Htt97Q variant was the longest poly(Q) tract that could be stably expressed, at least in their setting.

As research progresses, it will be interesting to see whether other yeasts are fruitfully used as HD models. For example, *Pichia pastoris* also provides many well-established genetic tools and is often the species of choice for heterologous protein expression (Gasser et al., 2013). Especially, the capability to reproduce human glycosylation patterns will become interesting (Gasser et al., 2013), regarding its involvement in HTT toxicity.

## Limitations and Considerations When Using Yeast as a HD Model

As for any model system, the benefits of handiness and cost-efficiency come at the expense of several disadvantages that need to be carefully taken into consideration when studying mHTT in yeast. In this chapter, we will name only the more general ones and omit those specific limitations that are subject to very particular questions.

One of the most obvious restraints of yeast for neurodegeneration research in general is the lack of multicellularity and inflammatory processes as seen in mammals at all levels. Indeed, many features of neurodegeneration depend on inter-cellular communication/interaction (Garden and La Spada, 2012), among them processes like neuroinflammation (Möller, 2010), and vesicle release/transport (Morton et al., 2001; Li et al., 2009). Highly specialized functions of neuronal cells cannot be fully studied in yeast cells, although basic features (e.g., endo- and exocytosis as rudimentary functions of vesicle transport and neurotransmitter release) may be present. In fact, this limitation may be advantageous for specific queries, as disease mechanisms can be studied isolated from each other without inter-tissue effects. Still, one must not dismiss the fact that HTT is expressed in all cells throughout the body, and pathological effects appear not exclusively in the brain. Alterations in peripheral tissues may sometimes be secondary to neuronal dysfunction, but have also been shown to be directly caused by expression in a specific tissue (van der Burg et al., 2009). Thus, although sometimes neglected, yeast may serve as a potent platform to study non-neurological and more general effects of mHTT.

Nonetheless, when studying neurodegenerative diseases in yeast, results should be interpreted with particular care if not validated in higher eukaryotes. Although many biochemical pathways and organelle functions are basically conserved in yeast, some show significant differences. Of note, yeast mitochondria lack a typical complex I and, instead, use three different, simpler NADH dehydrogenases. These are located externally (*NDE1* and *NDE2*) and internally (*NDI1*) to the inner mitochondrial membrane and do not show rotenone-sensitivity (Matus-Ortega et al., 2015). Additionally, none of the three NADH dehydrogenases present in yeast translocates protons, contrary to mammalian mitochondria (Matus-Ortega et al., 2015). Still, heterologous expression has shown significant functional overlap for the yeast NADH dehydrogenases in mammalian systems. For instance, expression of *NDI1* was sufficient to functionally replace complex 1 in cell culture (Yagi et al., 2006) and, moreover, extend lifespan and modulate energy metabolism of flies (Bahadorani et al., 2010; Sanz et al., 2010) as well as counteract detrimental effects of a Parkinson’s disease mouse model (Barber-Singh et al., 2009).

Large-scale yeast genetic screens with heterologous expression of human disease-relevant proteins are an appealing way to study molecular targets and identify potential disease-promoting pathways (Forsburg, 2001; Giorgini et al., 2005). However, genetic knockouts, for instance, may heavily influence cellular metabolism or modify expression levels, often leading to false-positive phenotypes. Thus, extensive low-throughput validation of screen results remains a prerequisite to rule out artifacts. Regarding HD yeast models, this specific problem can be tackled, for example, by simultaneously analyzing GFP signals when using high-throughput flow cytometry-based cell death analyses. If an apparent screen hit diminishing poly(Q) toxicity also lowers the expression level of HTT itself at some stage, it still might be a mechanistic way of action, but often is an unwanted artifact. In addition, the fact that mammalian HTT does not have a direct homolog in yeast, raises justifiable questions about protein handling, localization, and specification of interactions of heterologously expressed HTT. However, as outlined in the chapters before and hereafter, the translatability of findings in yeast models of HD into higher organisms strongly argues for some degree of specificity and, thus, also applicability of this simple organism for HD research. Further, similarities of human HTT, which is a relatively big and complex protein, with the yeast proteins Atg11p, Vac8p, and Atg23p, all important factors in the cytoplasm-to-vacuole targeting clearance pathway, have been noted (Steffan, 2010). This is in line with Ochaba’s recent suggestion, that HTT is a key factor in selective autophagy (Ochaba et al., 2014).

In many yeast models of HD, *GAL1*-promoter vectors are applied, which require the use of metabolically active galactose as an inductor for expression. The choice of carbon source in pre-induction medium (e.g., glucose, raffinose, and glycerol) is of utmost importance and can heavily influence the course of an experiment, as it changes (for instance) the mitochondrial status previous to the expression of HTT constructs. In fact, it is evident that the major mechanisms of mHTT toxicity may vary in distinct genetic backgrounds (Serpionov et al., 2017). Additionally, effects associated with mitochondrial function should be cross-validated in different strain backgrounds that are more suitable for mitochondrial studies (e.g., W303, YPH499/500, and D273-10B), since one of the most widely used strains (BY4741) has a known deficiency in respiratory competence (Gaisne et al., 1999). Moreover, the choice of culturing container and environment may also impact molecular processes (not only) related to mitochondria: oxygen availability across the culture depends on both the surface-to-volume ratio and actions to increase turbulences in the cultures (e.g., baffled flasks), and may heavily impact experiments (Bisschops et al., 2015). Finally, under specific circumstances, it might be useful to test a GFP-only vector as an additional control to low-Q-repeat HTT constructs (e.g., Htt25Q), in order to confirm non-toxicity of the actual control and exclude non-sequence-specific expression artifacts (Berglund et al., 2017).

The widely used *GAL1* promoter, which is activated by galactose and repressed by glucose, has very strong and acute expression, while the copper-inducible *CUP1* promoter is suitable for high expression profiles after the diauxic shift (the transition phase when the preferred carbon source gets depleted and metabolism is primed for the use of an alternative one). Expression via these strong inducible promoters causes rather acute toxicity, making them preferable models for screening of putative pharmacological and/or genetic interventions. However, metabolism-dependent systems might not be the model of choice when investigating metabolic or physiological aspects associated with the disease (Ocampo and Barrientos, 2008). Promoters that are constitutively active (e.g., *TEF1*, *SSA1*, and *GPD*) might as well be employed, but differ greatly in their expression profiles (Peng et al., 2015). Interestingly, recently developed synthetic hybrid promoters, bimodulars system composed of an enhancer element (tandem repeats or combinations of upstream activating sequences) and a core promoter, exhibiting new and enhanced expression and control features have not yet found their way into yeast HD research, but could lead to improved models soon (Peng et al., 2015). Generally, a system that allows continuous and controllable protein expression levels via a metabolically inactive inductor (e.g., non-metabolized β-estradiol) represents a great advantage at different levels, including the possibility to freely choose the carbon source, expression start, and control expression levels. Thus, the use of the above mentioned *TEF1-7* promoter model (Ocampo and Barrientos, 2008; Ruetenik et al., 2016) will likely improve the robustness of findings in yeast-based HD studies. This might be of special interest in setups requiring a more gradual and tunable expression instead of an acute expression that declines over time (as seen with the *GAL1*-expression system).

## Cytotoxic Mechanisms in HD Yeast Models

Despite its relative simplicity, yeast harbors a significant number of cellular pathways and factors relevant to human neurodegeneration and HD, including conserved mitochondrial regulation (Galluzzi et al., 2016), endoplasmic reticulum (ER) and ER-associated protein degradation (ERAD) biology (Duennwald and Lindquist, 2008), vesicle fusion (Li et al., 2009), endocytosis (Meriin et al., 2003), lysosomal/vacuolar mechanisms (Rajakumar et al., 2017), autophagy (Yin et al., 2016), lipid biology (Klug and Daum, 2014), RCD (Carmona-Gutierrez et al., 2010, 2018), oxidative stress (Sorolla et al., 2011), and cell cycle control (Bocharova et al., 2008). The high degree of conservation enables researchers to reliably model disease mechanisms in a highly controllable environment.

### Aggregation as an Intrinsic Feature of HD

One of the key features of neurodegenerative diseases is aggregation (to various degrees) of multiple intra- or extracellular proteins. In HD, aggregation of mHTT derives from an elongated poly(Q)-stretch in exon-1 (Taylor et al., 2002), which can also be observed in yeast models of HD (Krobitsch and Lindquist, 2000; Meriin et al., 2002; Ocampo and Barrientos, 2011; Kaiser et al., 2013). Recently, the ultrastructural architecture of a GFP-tagged human exon1-97Q construct was explored in yeast (Gruber et al., 2018). Both fibrillar and – to a major part – unstructured inclusions were found, while no detrimental interactions with cellular membranes were seen (Gruber et al., 2018). This stands at odds with previous findings of a similar construct forming amyloid-like fibrils in mammalian cells that interact with endomembranes, preferentially those of the ER (Bäuerlein et al., 2017). This ultrastructural discrepancy of basically similar mHTT constructs has yet to be fully put into biological context and needs further effort to be understood. However, this might also argue for the cellular context being highly relevant for aggregation and, thus, may provide another variable adding to the complexity of cell-specific effects by mHTT in human pathology. As Gruber et al. (2018) have argued, it is indeed conceivable, that the dissimilar chaperone systems of yeast and mammalian cells might be one of the reasons for these differences.

Commonly, yeast models of HD display largely cytosolic distribution of aggregates, although localization of aggregates into the nucleus dependent on yeast metacaspase YCA1 has been described (Sokolov et al., 2006). In line with data from mammalian cells, in yeast, this is accompanied by DNA cleavage, cytotoxicity, and cell cycle defects, which is reminiscent of the phenotype in human neurons (Sokolov et al., 2006; Zheng et al., 2017). Since different localization profiles of HTT aggregates have been described in yeast, this needs further clarification. It is likely that the contribution of the localization to cytotoxicity in yeast is subject to various variables, such as the properties of different constructs, timepoints, experimental setup, and aggregate-subtype. However, transition between or localization to different cellular subtypes is most likely not the sole major determent of toxicity.

Overall, after intense research, it is still not possible to fully capture the whole picture of the interplay between aggregation and toxicity. Still, from a wider point of view, the connection between aggregation processes *per se* and toxicity seems rather clear. There has been a paradigm shift, that the initially identified large aggregates caused by mHTT are not the major mediators of its toxicity. This might be attributed to the limitations of early microscopy. To sum up, it is likely, but not fully understood, that certain species other than the final form of large inclusion bodies cause mHTT-mediated cytotoxicity in yeast (and other models) (Bocharova et al., 2009).

The actual contribution of aggregates to neuronal cytotoxicity in humans has also yet to be fully elucidated. Although many studies suggest a correlation between aggregate formation and HD (Bates, 2003; Arrasate and Finkbeiner, 2012), the causal connection remains a matter of debate. A major reason for that is the fact that in human brain samples, neuronal aggregates are not necessarily overrepresented in those cells (medium spiny projection neurons) or tissues (striatum) that die the earliest or are affected the most (Gutekunst et al., 1999; Kuemmerle et al., 1999).

### Huntingtin Aggregation Can Be Modulated by Genetic and Pharmacological Means

Aggregation has been shown to be a (partially) specific process that depends on the poly(Q)-length and can be modified both by genetic and pharmacologic means. Prominent insights into mHTT aggregation were obtained by yeast studies, like the intensively studied involvement of chaperones in the aggregation process. Similarly, yeast has contributed to the identification and characterization of HD-relevant small molecule anti-aggregation agents, including the polyphenol (-)-epigallocatechin-3-gallate or the synthetic chemical compound C2-8 (see Chapter below) (Krobitsch and Lindquist, 2000; Zhang et al., 2005; Ehrnhoefer et al., 2006).

Interestingly, a recent study in yeast has shown different stages of aggregation for both Htt25Q and Htt103Q with significant alterations over time, including the surprising finding that (non-toxic) Htt25Q can form large aggregates as well (Xi et al., 2016). This argues for the concept that – if aggregation promotes toxicity – it is not by aggregation *per se*, but rather via a distinct population of aggregates. In this study, the authors argued for a correlation of mid-sized aggregates with cytotoxicity and could show that factors blocking toxicity of Htt103Q [including elimination of the poly(P) region] also changed the aggregation pattern, including a decrease of mid-sized and large aggregate populations (Xi et al., 2016). A large body of evidence indicates that Hsp40 chaperones can suppress poly(Q) aggregation, an observation already made as early as 2000 from yeast studies (Muchowski et al., 2000). Intriguingly, a recent yeast study revealed that the essential Hsp40 chaperone Sis1 is sequestered into mHTT aggregates, which subsequently compromises proteasomal degradation (Park et al., 2013). The importance of Hsp40 chaperones is further underlined by Drosophila (Kuo et al., 2013) and mouse (Popiel et al., 2012) studies, arguing for this type of chaperones being major players in suppressing poly(Q) aggregation and potential therapeutic targets. Moreover, recent literature shows that many endogenous proteins may be sequestered and, thereby, their function may be impaired by interactions with poly(Q) aggregates in yeast. Notably, additionally to a global reduction, mitochondrial proteins were found to be favorably sequestered by the aggregates, leading to changed mitochondrial morphology (Gruber et al., 2018).

Manifold genetic factors, especially chaperones (for example Hsp104) have been extensively linked to mHTT aggregation from yeast to rodents (Krobitsch and Lindquist, 2000; Lo Bianco et al., 2008; Lee and Goldberg, 2010; Cushman-Nick et al., 2013) (see also Chapter “Yeast as a Model Organism for Neurodegenerative Diseases”). Noteworthy, Hsp104 has also been implicated and studied as a therapeutic agent in other aggregate-associated neurodegenerative diseases, such as Alzheimer’s and Parkinson’s disease (Vashist et al., 2010). Although metazoan lack Hsp104 orthologs, diverse beneficial effects, including lifespan-extension of a HD mouse model (Vacher et al., 2005), have been achieved with expression of this chaperone.

A recent study has also implicated increased glycation (achieved by elevated levels of methylglyoxal, the most potent glycation agent) as a mechanism aggravating Htt72Q and Htt103Q aggregation in yeast, Drosophila and human cell lines (Vicente Miranda et al., 2016).

### To Clear or Not to Clear? Autophagy and HD in Yeast

Macroautophagy (hereafter referred to as autophagy) is a well-conserved intracellular recycling program and, together with the proteasome, one of the major degradation and proteostatic processes. Several subtypes have been described, that can eliminate damaged or overdue organelles, aggregates, or other cellular content (Yin et al., 2016). Autophagy has been heavily implicated in HD pathology and discussed as a therapeutically targetable pathway to clear aggregates and improve cellular homeostasis (Wong and Cuervo, 2010; Lin and Qin, 2013). Thereby, yeast has played a primary role in characterizing the autophagic process in general (Ohsumi, 2014; Zimmermann et al., 2016) and the autophagic machinery in particular, including the ATG genes, which are essential for autophagic functioning. In yeast, autophagy is needed for the delivery of aggregates to vacuoles (Chuang et al., 2016) and mutant strains Δ*atg8*, Δ*cue5*, and *rsp5-2*, which have autophagy and/or ubiquitylation deficiencies are more sensitive to poly(Q)-expression (Lu et al., 2014). In the same study, yeast experiments revealed the ubiquitin-binding CUE-domain protein Cue5 as an important player in the clearance of aggregates and selective autophagy (Lu et al., 2014). Interestingly, a recent yeast study has corroborated these findings by showing that deletion of the ubiquitin-interacting protein Dsk2 alters the Htt103Q aggregation profile toward a more dispersed distribution of smaller aggregates and Δ*dsk2* strains show elevated sensitivity to poly(Q) cytotoxicity (Chuang et al., 2016). Overall, it seems that HTT clearance in yeast heavily relies on both the ubiquitin proteasome system and autophagy, with many factors involved and time-dependent differences that must be taken into consideration. Moreover, a chaperone network likely contributes and coordinates the formation of inclusion bodies, which are then subject to autophagic degradation (Higgins et al., 2018). Concurringly, strains having defects in the chaperone system (Δ*sse1*, Δ*fes1*, and Δ*ydj1*) show reduced colocalization of HTT and Atg8p, an essential autophagic adapter protein (Higgins et al., 2018).

Elevated autophagic levels increase cellular viability and decrease soluble and aggregated forms of HTT in cell culture, Drosophila and mouse models (Rubinsztein, 2006). Perceptibly, the autophagy-inducer rapamycin, a well-known inhibitor of TOR (target of rapamycin; negative regulator of autophagy), as well as rapamycin-analogs exert protective effects in various HD models (Ravikumar et al., 2004; Sarkar et al., 2007). In other cases though, mHTT seems to increase autophagy in different species (Sarkar and Rubinsztein, 2008). Notably, mTOR activity is reduced (hence autophagy upregulated) by sequestration into HTT aggregates in mouse models and human brain samples (Ravikumar et al., 2004). Nevertheless, increased autophagy in these cases may represent a cellular reaction rather than a causal toxicity mechanism (Carmona-Gutierrez et al., 2018; Galluzzi et al., 2018). We therefore argue for the intensified and reinforced use of yeast as a model system to elucidate the connection and therapeutic potential of autophagy and HD.

A physiological role of native HTT in autophagy has recently been suggested: the weak similarity of HTT to yeast Atg11, Atg23, and Vac8 has led to the hypothesis that HTT might act as a scaffold protein in selective autophagic processes (Steffan, 2010; Ochaba et al., 2014). Still, as mentioned above, the complete native function of HTT remains elusive. The ratio of wild-type HTT and mHTT seems to play a role in the disease manifestation, at least in mice (Leavitt et al., 2001). Certainly, in Htt25Q/103Q coexpression experiments in yeast, the low-Q variant seemed to increase the solubility of Htt103Q, which reduced oxidative stress and toxicity (Saleh et al., 2013). This, however, remains open to debate and needs further studies, as opposite findings have been described in the literature. However, in this particular study, the authors found an expression-dependent dynamic: highly expressed Htt25Q enhanced the solubility of Htt103Q, while at lower levels, it was preferentially sequestered to aggregates itself (Saleh et al., 2013). Therefore, it could be that the interaction of wild type und mHTT follows a complex dynamic, which could explain the contradictory reports.

### Discovering Further Toxicity Mechanisms in Yeast

The possibility of feasible genetic screens has been consistently employed upon modeling HD in yeast. For instance, an early study identified 52 genetic enhancers of toxicity, which were predominantly related to stress response and protein folding pathways (Muchowski et al., 2000; Willingham et al., 2003). This is in line with numerous studies on different stress signaling pathways affected by HD (Duennwald, 2015). As an example, the heat shock response (HSR) via the transcription factor Hsf1 is impaired by elongated poly(Q) HTT with extensive two-sided interactions and HSR proteins (e.g., Hsp40 and Hsp70) modulating aggregation and toxicity (Duennwald, 2015) (see above). Furthermore, it is well established that mHTT impairs ER stress response, leading to accumulation of misfolded proteins in the ER and activation of the unfolded protein response (Kouroku et al., 2007; Matus et al., 2008). In line, yeast cells expressing mHTT display an acute and specific ER stress response that is to some extent the consequence of essential ERAD components (Npl4p, Ufd1p, and p97) being trapped in HTT aggregates (Duennwald and Lindquist, 2008). In that study, however, HSR was not activated by elongated poly(Q) expression (Duennwald and Lindquist, 2008). Both pathways (HSR and ERAD) might comprehend druggable targets in the future. Of note, the Golgi stress response is also compromised in HD (Sbodio et al., 2018), and HTT has been implicated in an immediate type of stress response including so-called huntingtin stress bodies, which might be defective in HD (Nath et al., 2015). Another genome-wide knockout screen could show that polyploidy represents a resistance mechanism against poly(Q) toxicity (Kaiser et al., 2013). In that study, haploid cells were more susceptible to Htt56Q-induced growth defects and proteins within the septin ring (e.g., Cdc10, Shs1) seemed to mislocalize under poly(Q)-stress. Twenty-eight out of the 38 toxicity-modulating screen hits also had an impact on the Htt103Q model, and tetraploid strains (e.g., PY5006, PY5007) were resistant to poly(Q) toxicity (Kaiser et al., 2013). Though, the implications of these findings for human HD pathology have yet to be investigated.

### Mitochondria as Central Organelles in HTT Toxicity

Persistent ER stress response is accompanied by oxidative stress and mitochondrial impairment, resulting in cell death (Haynes et al., 2004). Mitochondrial defects in human HD pathology, especially in neurons with high energy demand, are indeed the consequence of multiple perturbations caused by mHTT (Braun, 2012; Farshbaf and Ghaedi, 2017). Similarly, in yeast, mHTT expression leads to detrimental respiratory defects, impairs complex II and III, enhances production of reactive oxygen species and alters mitochondrial morphology (Solans et al., 2006). Furthermore, longer poly(Q) lengths of 72 and 103 glutamines seem to increase the fragments’ tendency to associate with mitochondria and impair mitochondrial membrane potential (Ocampo et al., 2009). Congruently, activation of mitochondrial biogenesis (through overexpression of *HAP4*, a regulator of nuclear-encoded mitochondrial genes) counteracts these effects in yeast and Drosophila (Ocampo et al., 2009; Ruetenik et al., 2016). In line with this, poly(Q) toxicity and oxidative stress are aggravated in respiring yeast cells (Sorolla et al., 2011). A pathological feature of HD patients that can also be observed in yeast are metabolic alterations (Aziz, 2007; Papsdorf et al., 2015). In a yeast model, poly(Q) toxicity could be connected to iron metabolism and mitochondria (Papsdorf et al., 2015). Indeed, elevated iron levels have been reported in HD by some studies, but how they affect respective neurons and brains remains controversially discussed (van den Bogaard et al., 2013; Muller and Leavitt, 2014).

### Endocytic Processes, Prions, and the Intracellular Localization of Aggregates Are Key-Determinants of Toxicity

In mouse models and human samples of HD, components of the vesicle fusion machinery, endocytosis, and exocytosis are negatively affected (Velier et al., 1998; Morton and Edwardson, 2001; Morton et al., 2001; Trushina et al., 2006; Li et al., 2009). Using yeast, two recent studies have analyzed the involvement of ribosomal quality control and type-1 myosin-dependent endocytosis systems, respectively (Berglund et al., 2017; Zheng et al., 2017). Zheng et al. (2017) showed a positive correlation between Htt103Q nuclear localization and cytotoxicity and could identify several factors involved in the nucleocytoplasmic distribution of Htt103Q. Deletions of the ubiquitin ligase *LTN1* or *RQC1*, a component of the ribosome quality control complex (RQC), promoted nuclear localization via the nuclear pore complexes. This transport mechanism involves the v-SNARE-binding protein Btn2, the overexpression of which blocked nuclear accumulation of Htt103Q (Zheng et al., 2017). Interestingly, Btn2 has been shown to exert anti-prion properties (Wickner et al., 2014), which – given the dependency of mHTT toxicity on Rnq1p and its prion status in yeast – is of special interest and should be further explored. Interestingly, out of six identified suppressors of Htt103Q toxicity (including Hsp40 chaperone Sis1) in an overexpression screen, three possessed Q-rich, prion-like domains, namely Gts1, Mcm1, and Nab3 (Ripaud et al., 2014). The authors found that these proteins could modulate aggregation to more non-toxic forms, preventing the aggregates from interacting with endogenous proteins and increasing the interactions with chaperones (Ripaud et al., 2014). This puts extra emphasis on the importance of prion biology for HD. Additionally, the interaction of Q-rich proteins with HTT and the possible modulation of toxicity thereby, might be an important line of research to understand basic toxic mechanisms of HD.

In a genome-wide screen for poly(Q) toxicity, Berglund et al. (2017) discovered that the 25Q version is toxic in strains with defects in actin- and type-1 myosin-dependent endocytosis. This enhanced toxicity of the soluble HTT variant was abolished in cells expressing the aggregation-prone 103Q version (Berglund et al., 2017). It is tempting to speculate that this may contribute to positive selection of longer poly(Q) tracts. Thus, although yeast lacks a vesicular system as needed for the modeling of neurotransmission defects, basic processes, and alterations caused by mHTT still might be studied fundamentally. Interestingly, endocytosis was reported to play a central role in pathological mechanisms of amyotrophic lateral sclerosis models in yeast and flies (Liu et al., 2017; Leibiger et al., 2018). Hence, it will be also interesting to see in the future, how dysfunctional endocytic processes may be incorporated into the complex picture of cytotoxic mechanisms in HD.

### Is RCD Implicated in HTT Toxicity in Yeast?

Neuronal loss (partly) due to RCD is a shared feature of many neurodegenerative diseases (Vila and Przedborski, 2003). Studies on neuronal loss in HD have reported signs of apoptotic and, more recently, also necrotic cell death (Portera-Cailliau et al., 1995; Sawa et al., 2003; Mao et al., 2016). Yeast has a long-standing history in RCD research, including the modeling of mammalian cell death processes (Madeo et al., 1997; Carmona-Gutierrez et al., 2010, 2018) in general and neurodegenerative mechanisms in particular (Pereira et al., 2012). Indeed, prominent RCD subroutines have been characterized, including apoptosis and regulated necrosis. In addition, core components orchestrating RCD are highly conserved (Carmona-Gutierrez and Büttner, 2014; Carmona-Gutierrez et al., 2018). Still, data on RCD mechanisms/pathways in yeast HD models remain rather scarce.

In conclusion, aggregation, elevated ER stress levels, autophagy, and RCD are closely connected (Kouroku et al., 2007; Matus et al., 2008), and yeast incorporates many conserved pathways and convenient techniques to further elucidate the interplay of these neurodegenerative hallmarks.

## Yeast as a Screening Platform for Drug Discovery

**FIGURE 1:**
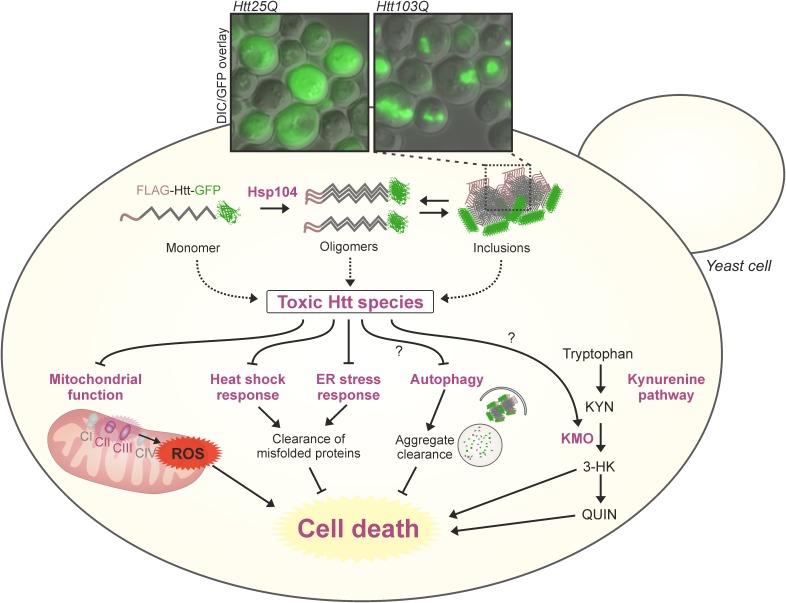
Sights and mechanisms of mHTT toxicity in yeast models of HD. Multiple sources and modulatory pathways of mHTT toxicity have been identified in different models of HD in yeast (dysfunctional mitochondria, aggregation, altered stress responses, kynurenine pathway, and more). The combination of toxic effects leads to increased cell death. The microscopy inset shows pictures of typical aggregation of GFP tagged exon 1-poly(Q) constructs in a widely used *S. cerevisiae* model of HD (Meriin et al., 2002), 24 h after galactose-induction of chronologically aged yeast cells. KMO, kynurenine 3-monooxygenase; ROS, reactive oxygen species.

**FIGURE 2:**
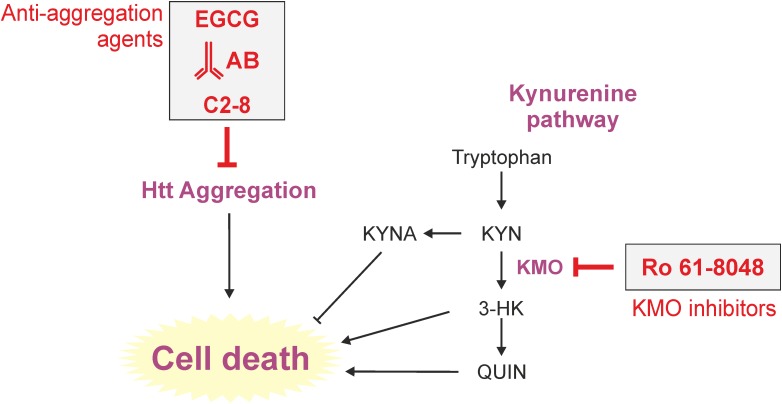
Therapeutic approaches to tackle toxicity and/or aggregation in yeast models of HD. Yeast has long served as a tool to identify pharmacological agents against HD. Here, important processes (aggregation and kynurenine pathway) of HD toxicity in yeast are depicted. Prominent approaches previously used in yeast are the application of the polyphenol epigallocatechin-3-gallate (EGCG), intrabodies or the small molecule C2-8, all in order to reduce intracellular aggregation load. The KMO inhibitor Ro 61-8048 was used to modulate the course of tryptophan degradation via the kynurenine pathway, which is heavily implicated in HD, leading to less cell death. AB (here intrabody), EGCG (epigallocatechin-3-gallate), KMO (kynurenine 3-monooxygenase).

One of the most crucial advantages of modeling human diseases in yeast is the possibility of time- and cost-efficient high-throughput screens (Barberis et al., 2005). Beyond the feasibility of uncovering mechanistic insights via genetic screens (see above), yeast offers great potential for drug discovery, also targeting neurodegenerative diseases (Tardiff et al., 2013; Menezes et al., 2015; Park et al., 2016; Porzoor and Macreadie, 2016; **Figures 1**, **2**). Studies in this unicellular eukaryote have led, for example, to the discovery of the anti-aging agents spermidine (Eisenberg et al., 2009) and resveratrol (Howitz et al., 2003). Follow-up and validation studies have often proven the translational potential of yeast findings to higher model organisms (**Table 2**).

**Table 2:** Basic drug findings in yeast models of HD and their translational validation in cell culture, *Drosophila melanogaster* and rodents.

Compound	Effect	Validation		
		**Cell culture**	**Drosophila**	**Rodents**
		
Intrabodies (V_L_12.3, MW7, C4 sFv, Happ1, Happ3)	V_L_12.3 reduces aggregation and toxicity (Colby et al., 2004)	V_L_12.3, MW7, Happ1, and Happ3 reduce toxicity and aggregation of HDx-1 (Khoshnan et al., 2002; Southwell et al., 2008)	C4 sFv reduces aggregation and cellular toxicity (Wolfgang et al., 2005)	Happ1 improves neuropathology in transgenic and lentiviral HD mouse models (Southwell et al., 2009) Intrabodies act via different mechanisms and partially show adverse effects in HD mouse models (Southwell et al., 2009; Butler et al., 2014)
C2-8	Aggregation inhibition (Zhang et al., 2005)	Long-term inhibition of elongated poly(Q) aggregation (Zhang et al., 2005)	Dose-dependent suppression of pathogenesis (Zhang et al., 2005)	Reduction of mHTT aggregation, but no improvement of motor dysfunction of striatal neurodegenerative pathology in R6/2 HD transgenic mice (Wang et al., 2013)
Ro 61-8048	Inhibition of kynurenine 3-monooxygenase (KMO) (Giorgini et al., 2005)	–	Inhibition of KMO in a Drosophila model of HD (Campesan et al., 2011)	Inhibition of KMO in a mouse model of HD (Zwilling et al., 2011)
Epigallocatechin-3-gallate (EGCG)	EGCG is a suppressor of mutated huntingtin aggregation and toxicity (Ehrnhoefer et al., 2006)	Protective effect on rat hippocampal neuronal cells (Menard et al., 2013)	Increases fitness and lifespan in Drosophila (Wagner et al., 2015)	EGCG extends lifespan of rats (Niu et al., 2013)
Actinomycin D	Aggregation inhibition (Walter et al., 2014)	Suppresses aggregation of elongated poly(Q) in mammalian cells (Walter et al., 2014)	–	–

*EGCG, epigallocatechin-3-gallate; KMO, kynurenine 3-monooxygenase*.

### Drug Candidate Discovery Through Pharmacological Screens in Yeast

In fact, the use of commercially available libraries and pharmacological approaches to find toxicity-modulating substances were already applied early on in yeast HD research (**Figure 2**). In 2004, it was shown that the intrabody V_L_12.3 could reduce aggregation and toxicity in a yeast model with integrated exon-1-Htt25Q/72Q constructs (Colby et al., 2004). Some of these beneficial effects could be later corroborated in cell culture (Southwell et al., 2008) and lentivirus mouse models (Southwell et al., 2009) of HD. However, adverse effects were shown too in a transgenic HD mouse model (Southwell et al., 2009; Butler et al., 2014). Irrespectively, the study helped strengthening the intrabody-concept as a therapeutic approach against HD and other intrabodies (e.g., MW7, C4 sFv, Happ1) against HTT have been tested (Wolfgang et al., 2005; Southwell et al., 2009; Messer and Joshi, 2013).

Zhang et al. (2005) used a yeast-based approach including 16,000 substances to identify and further validate an aggregation-inhibiting compound, C2-8 (N-(4-bromophenyl)-3-{[(4-bromophenyl)amino]sulfonyl}benzamide), in Drosophila and mouse tissue. Several chemical compounds showed anti-aggregation properties in yeast, while the structural analog of C2, C2-8, did so in a dose-responsive manner and with low IC_50_ values (50 nM). They found that the compound’s aggregation inhibition greatly relies on cellular systems, arguing for cellular targets, and that it prominently affects the polymerization/growth of aggregates, rather than their nucleation/initiation. For screening, a Δ*erg6* mutant strain with increased membrane permeability was employed. Primary hit selection was based upon photometric assessment and/or eGFP fluorescence, which was then microscopically validated. The substance has ultimately led to successful pre-clinical studies in HD mouse models, proving the concept of yeast as an initial screening tool for toxicity modulators (Chopra et al., 2007; Wang et al., 2013). It should be noted that in this study, the use of an Δ*erg6* mutant obeyed the rationale that in yeast, specific cell wall and drug transporters represent obstacles for any non-endogenous substance. In order to ease the transition of drugs into or slow the secretion out of the cell, strains deficient in multi-drug transporters (Δ*pdr5*) (Walter et al., 2014) or increased membrane permeability (Δ*erg6*) (Zhang et al., 2005), for example, are valid approaches, respectively. Yeast ABC (ATP binding cassette transporter) proteins, including pleiotropic drug resistance (PDR; e.g., *PDR5*) and multidrug resistance subfamilies, have been implicated in detoxification of exogenous substances. On the other hand, the deletion of such machineries may impact cellular physiology at other levels, as efflux pumps are important for mitochondrial homeostasis, secretion processes, organelle biogenesis, stress response, and physiological uptake of substances from the culturing environment (Bauer et al., 2003; Schüller et al., 2003). Thus, studying tested compounds in a transporter-unmodified yeast strain represents a physiologically more intact setting and can also provide useful insights into the bioavailability of drugs in an eukaryotic system (Zimmermann et al., 2018).

### The Discovery of the Kynurenine Pathway

Using the same model as Zhang et al. (2005) and Giorgini et al. (2005) performed a loss-of-function genomic suppressor screen and identified an enzyme (kynurenine 3-monooxygenase, KMO; in yeast *BNA4*) in the kynurenine pathway of tryptophan degradation, the deletion of which abolished Htt103Q toxicity. Interestingly, KMO had been previously associated with human HD pathology (Beal et al., 1990, 1992; Pearson and Reynolds, 1992; Stoy et al., 2005). The kynurenine pathway is now well-studied and discussed as a drug target for HD (Thevandavakkam et al., 2010; Wild and Tabrizi, 2014; Jacobs et al., 2017). In their original study, Giorgini et al. (2005) already showed a partial amelioration of growth defects in Htt103Q-expressing yeast cells treated with Ro 61-8048, a small molecule inhibitor of KMO. KMO inhibition leads to an altered product and intermediate profile of tryptophan degradation, reducing cellular stress and cell death. Further on, KMO inhibition has been extensively approached pharmacologically and chemically in pre-clinical rodent and Drosophila HD models (Campesan et al., 2011; Zwilling et al., 2011) and might be tested in early human trials in the near future.

### Natural Substance Screens With Yeast HD Models

Shortly after the above-mentioned studies, Ehrnhoefer et al. (2006) screened a natural compound library using a W303 strain containing integrated GFP-tagged exon-1-HTT with either a 25Q or 72Q C-terminal stretch. They could elegantly show that the polyphenol (-)-epigallocatechin-3-gallate (EGCG) is a potent suppressor and modulator of mHTT aggregation and toxicity *in vitro* and *in vivo* in yeast and fly models of HD. Interestingly, the substance seems to inhibit aggregation *per se* in yeast cells, but also promotes the formation of larger aggregates *in vitro*. The substance, which is a major bioactive component in green tea, has become a promising candidate for healthy aging and promotes lifespan extension in worms, flies, and rodents (Zhang et al., 2005; Niu et al., 2013; Wagner et al., 2015; Chu et al., 2017; Pallauf et al., 2017). EGCG has been thoroughly examined in the context of neurodegeneration and several independent studies have shown its anti-aggregation properties (Avramovich-Tirosh et al., 2007; Hudson et al., 2009; Liu et al., 2015; Debnath et al., 2016). Animal and human studies (including phase 2 trials) have already been performed with EGCG, though the applicability in humans has been questioned (Mereles and Hunstein, 2011; Mähler et al., 2013).

Walter et al. (2014) also screened natural compounds against HD toxicity and aggregation. They successfully identified actinomycin D as a potent aggregation inhibitor. The substance was applied in nanomolar concentrations and increased the levels of certain heatshock-proteins (e.g., *HSP104*, *HSP70*, and *HSP26)*. The group could further transfer their findings to mammalian cell culture, implying conserved actions of actinomycin D. Noteworthy, the drug actinomycin D (or dactinomycin) has many approved medical uses and could become an exciting drug lead in HD research.

## Concluding Remarks

*Saccharomyces cerevisiae* has a long-standing history in basic and applied research (Barnett, 2003), and the areas in which it can be used continue to expand in content and number. Indeed, researchers worldwide still rely on its robustness, handiness, applicability and scientific relevance. It may seem bold to study neurodegeneration in a unicellular fungus, but the richness of findings obtained from yeast studies and their translational potential have clearly proven a point. Although many aspects of neurodegenerative processes lie beyond the capacity of yeast models, numerous important features of age-related and neurodegenerative diseases in general and HD in particular can reliably be modeled and studied in yeast.

In this review, we have given a glimpse of the extensive possibilities and significance of yeast for HD research by summarizing some of the most significant insights gained from yeast experiments. We are aware that this short overview does not grasp every aspect of HD research conducted in yeast, but it conveys the convincing potential of yeast as a tool to help both understand and counteract HD. During the last few years, the knowledge about the disease has developed especially fast (McColgan and Tabrizi, 2017), and we expect an accelerated pace of scientific outcome that is relevant for HD patients in the near future.

### What Can Be Expected From Yeast in the 21st Century?

With rapid digitalization and big data science becoming increasingly important in basic research, the possibilities of yeast as an *in vivo* quasi “programmable” cell will continue to be of valuable importance. Especially regarding big data production, network analyses and systems biology, there is yeast, and in particular *S. cerevisiae*, at the forefront of data generation and development. The feasibility of performing complex and powerful screens has already been remarkably proven in the past decades. However, we believe that the potential is not exhausted. Modern screening systems already allow the automatic inoculation, mating, dilution, microscopy, and assessment of various parameters in large strain collections. In the near future, we expect to see various screening applications that employ single-cell-techniques, stratifying data, and zooming into individual cell biology. Small-scale omic-approaches as well as upscaled production suites will also likely contribute to pathway finding and therapeutic production, respectively.

Still, the relevance of yeast is not restricted to a screening tool. Instead, the progressing humanization of yeast strains and the methodologically well-engineered manipulation techniques will yield possibilities for systems biology and especially synthetic biology that go beyond the current state of research. Sophisticated, novel methods, and engineered strains will also facilitate research on neurodegenerative diseases.

In that respect, we are sure that yeast approaches will further accompany the research on HD and continue to provide assistance in deciphering fundamental questions.

## Author Contributions

SH, FM, and DC-G wrote the manuscript and designed the review. KK, AZ, MB, TP, and MP were involved in the generation of the table and figure and critically revised the manuscript for important intellectual content. All authors contributed to the conception of this review article.

## Conflict of Interest Statement

The authors declare that the research was conducted in the absence of any commercial or financial relationships that could be construed as a potential conflict of interest.
